# Absolute Enumeration of Probiotic Strains *Lactobacillus acidophilus* NCFM^®^ and *Bifidobacterium animalis* subsp. *lactis* Bl-04**^®^** via Chip-Based Digital PCR

**DOI:** 10.3389/fmicb.2018.00704

**Published:** 2018-04-11

**Authors:** Sarah J. Z. Hansen, Wesley Morovic, Martha DeMeules, Buffy Stahl, Connie W. Sindelar

**Affiliations:** ^1^Probiotic Development, DuPont Nutrition & Health, Madison, WI, United States; ^2^Genomics and Microbiome Sciences, DuPont Nutrition & Health, Madison, WI, United States

**Keywords:** probiotic, enumeration, *Bifidobacterium animalis* subsp. *lactis* Bl-04, *Lactobacillus acidophilus* NCFM, chip-based digital PCR, plate count, propidium monoazide

## Abstract

The current standard for enumeration of probiotics to obtain colony forming units by plate counts has several drawbacks: long time to results, high variability and the inability to discern between bacterial strains. Accurate probiotic cell counts are important to confirm the delivery of a clinically documented dose for its associated health benefits. A method is described using chip-based digital PCR (cdPCR) to enumerate *Bifidobacterium animalis* subsp. *lactis* Bl-04 and *Lactobacillus acidophilus* NCFM both as single strains and in combination. Primers and probes were designed to differentiate the target strains against other strains of the same species using known single copy, genetic differences. The assay was optimized to include propidium monoazide pre-treatment to prevent amplification of DNA associated with dead probiotic cells as well as liberation of DNA from cells with intact membranes using bead beating. The resulting assay was able to successfully enumerate each strain whether alone or in multiplex. The cdPCR method had a 4 and 5% relative standard deviation (RSD) for Bl-04 and NCFM, respectively, making it more precise than plate counts with an industry accepted RSD of 15%. cdPCR has the potential to replace traditional plate counts because of its precision, strain specificity and the ability to obtain results in a matter of hours.

## Introduction

Total enumeration of viable bacteria is a key metric in probiotic industrial science used to ensure strain-specific health benefits, per the definition of probiotics (Hill et al., 2014). This has typically been assessed by the microbial growth on media that selects for target phenotypic and metabolic characteristics. The practice of obtaining colony forming units (CFU) by plating has been used since the late 19th century and is the current standard for enumeration in the probiotic industry (Davis, 2014) with publications from the International Organization of Standardization (ISO) and United States Pharmacopeia (USP) (United States Pharmacopeial Convention, 2015). However, there is no single culture-based methodology that is applicable to all probiotic organisms because there is considerable variability between species and strains in their response to plating procedures (Davis, 2014).

Another detection method, flow cytometry (FCM), has gained prominence in industry for the enumeration of cultures based on the technology to sort viable from dead cells by using a combination of cell membrane intercalating dyes and quantifying cellular physiological parameters with a laser detection apparatus (Bunthof et al., 2001). However, together the genomics era and the use of molecular tools like polymerase chain reaction (PCR) have revolutionized strain-specific microbial detection resolution to unique insertions/deletions (INDELs) or single nucleotide polymorphisms (SNPs) in DNA sequence (Mullis and Faloona, 1987; Lomonaco et al., 2015; Ricchi et al., 2017). Taken further, quantitative PCR (qPCR) combines detectable DNA-intercalating dyes like SYBR^®^ Green/EvaGreen (Masco et al., 2007; Kramer et al., 2009) or fluorophores on TaqMan^®^ probes (Heid et al., 1996; Malinen et al., 2003) to create standard curves from known quantities of starting template, which then can be used to correlate unknown quantities. Recently, next generation sequencing (NGS) efforts have estimated relative abundances of probiotics based on 16S rRNA gene profiling (Morovic et al., 2016) and metagenomics analysis (Patro et al., 2016). These methods all quantify microbes using technologies that also have limitations in crucial criteria.

The ideal method for enumerating bacteria should present a cost-effective and rapid means to accurately determine absolute levels of viable cells with resolution to the strain level. While CFU enumeration is a simple process, it lacks the speed and resolution of molecular technologies. Presently, the industry-accepted variability range of CFU methods is 20–30% or more (de Ruig, 1996; Corry et al., 2007; Jongenburger et al., 2010) or 10–15% RSD (standard deviation divided by the mean), and time to results is often 2–5 days of incubation time. Furthermore, the use of select antibiotics or specific media ingredients may allow differentiation between different species in some cases (Dave and Shah, 1996; Temmerman et al., 2003; Champagne et al., 2011; Oberg et al., 2011; Davis, 2014) but differentiating highly clonal strains is impossible unless there are visible phenotypic differences such as colony morphology or cell shape (Shah, 2000; Galat et al., 2016). Flow cytometry is an ISO/IDF method (ISO 19344|IDF 232, 2015) that can distinguish viable cells using quenching dyes that separately detect dead and damaged cells. While testing time is relatively short (within 8 h), and has less variability of 10%RSD (unreported data), the resolution of this test cannot distinguish differences in genotype (Amor et al., 2007). While qPCR can target genetic identity, its limitations require enumeration to be calculated from a relative standard curve. The qPCR workflow requires purified DNA as a sample template to avoid intra- and extracellular inhibitors. Methods and kits are publicly available for genomic DNA extraction, but there are many that do not effectively isolate 100% of the DNA present (McOrist et al., 2002). PCR amplifies extracellular DNA, which requires additional dyes like Propidium Monoazide (PMA) as a pre-treatment to differentiate viable cells from damaged (Nocker et al., 2007b; García-Cayuela et al., 2009; Kramer et al., 2009; Desfossés-Foucault et al., 2012; Fittipaldi et al., 2012; Dinu and Bach, 2013; Nkuipou-Kenfack et al., 2013; Barbau-Piednoir et al., 2014; Codony et al., 2015; Leifels et al., 2015; Erkus et al., 2016). Finally, NGS is much more expensive and requires trained bioinformatics support compared to the above methods and generates relative abundance of communities, albeit with an enormous sampling depth.

The manufacture of probiotic cultures often includes lyophilization post fermentation, prior to shipment. This puts them in a non-metabolic state causing suspension of most cellular functions until introduction into a life-supporting environment (Zárate and Nader-Macias, 2006; Kharchenko et al., 2017). Logistical handling can therefore have a detrimental effect on dormant bacteria due to stressors including heat and relative humidity (Poddar et al., 2014; Tripathi and Giri, 2014; Kolaček et al., 2017). Furthermore, products are often formulated in blends of multiple probiotic bacterial strains, which can confound tests that cannot discern between species or strains. Arguably, when highly similar organisms, such as different strains of *Bifidobacterium animalis* subsp. *lactis* or *Lactobacillus acidophilus* (Barrangou et al., 2009; Briczinski et al., 2009; Stahl and Barrangou, 2013), are combined they will not be discernible without genotypic assays. Products are often blended together with numerous excipients like flavorings, enzymes, and/or prebiotics which can inhibit the different tests for enumeration (Champagne et al., 2011). Taken together, probiotic products present challenges to enumeration technology that are not answered completely by any one traditional or molecular-based method.

**FIGURE 1:**
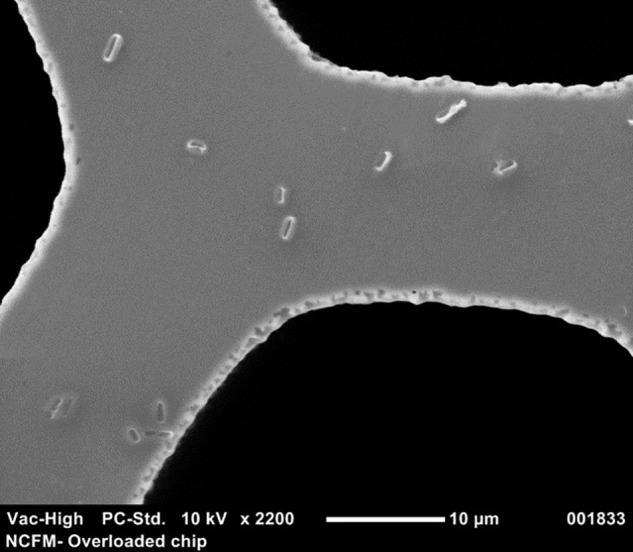
Scanning electron microscope picture at 2200× of a cdPCR chip that has been overloaded with culture to compare the size of single intact NCFM cells to wells.

Chip-based digital PCR (cdPCR) is a method of dividing and distributing microdilutions of sample template DNA across chips containing 20,000 wells (Ottesen et al., 2006; Zhang and Xing, 2007; Sanders et al., 2011; Li et al., 2016). When target template is present in a well, the amplicons from end-point PCR will fluoresce using TaqMan^®^ chemistry and result in a positive signal as read by a fluorimeter. The number of positive to negative signals on a chip is calculated through Poisson statistics to result in absolute copies per μL (Jacobs et al., 2017). This process has been validated to quantify the relative abundance of mutations that cause human cancer and disease (Kinz et al., 2015; Sefrioui et al., 2015), clinical viruses (Hayden et al., 2016), and pathogens (Blaya et al., 2016), but its efficacy has not been tested on probiotics to the best of our knowledge. Thus, we obtained a QuantStudio^®^ 3D Digital PCR system (Life Technologies, Carlsbad, CA, United States) to qualify probiotic cultures with cdPCR. We hypothesized that the size of the chip wells (**Figure 1**) and freedom from constraints of qPCR would allow for absolute bacterial quantification of intact cells, without prior full genomic DNA (gDNA) purification. This manuscript details the process development using cdPCR for absolute quantification of two common probiotic strains *L. acidophilus* NCFM and *B. lactis* Bl-04 both individually and in multi-strain material.

## Materials and Methods

### Bacterial Strains, Growth, and Isolation of DNA

Cell cultures of *L. acidophilus* NCFM and *B. animalis* subsp. *lactis* Bl-04 were grown in de Man-Rogosa-Sharpe (MRS) broth (BD Difco, Sparks, MD, United States) with 0.05% cysteine (Sigma-Aldrich, St. Louis, MO, United States) at 37°C for 18 h under anaerobic conditions. Genomic DNA (gDNA) used for qPCR was isolated using a Roche High Pure Template kit (Roche, Basel, Switzerland). gDNA for the validation of primers and probes and for viability detection was isolated using the DNeasy Tissue and Culture DNA Isolation Kit (Qiagen, Hilden, Germany). Commercially manufactured freeze dried culture was used in the application experiments to assess multiplex capabilities, as well as a final comparison to plate count enumeration. Dilution series of gDNA, overnight and lyophilized cultures were made using 1X Tris-EDTA pH 8.0 (1X TE, Thermo Fisher, Wilmington, DE, United States) to avoid DNA degradation.

### Scanning Electronic Microscopy

For a good visualization, samples were overloaded using 20 μl of a 10-fold dilution from an 18-h culture of NCFM which was applied to the QuantStudio^®^ 3D dPCR V2 20K chip using the QuantStudio 3D digital PCR chip loader (Life Technologies, Carlsbad, CA, United States). The sample was allowed to dry before gold sputter coating (Cressington 108, Ted Pella, Inc., Redding, CA, United States). The chip was then inserted into the scanning electronic microscope (Neoscope JCM-5000, McCrone Microscopes, Westmont, IL, United States) and images were captured at 2200× to allow for the visualization of the bacteria size comparative to the wells of the chip.

### Assay Design and Optimization

Primers and probes were designed to differentiate the target strains against other strains of the same species using known genetic deletions. For *B. lactis* Bl-04, an assay was designed using a 54 base pair (bp) deletion in a long-chain fatty-acid-CoA ligase gene (Briczinski et al., 2009). For *L. acidophilus* NCFM, an assay was designed around a 415 bp deletion in an ABC-type multidrug transporter in *L. acidophilus* La-14 (Altermann et al., 2005; Stahl and Barrangou, 2013). Sequences of both deletions were analyzed in Geneious v. 6.1.8 (Biomatters, Auckland, New Zealand) and oligos were designed using the *Primer3* plugin (Koressaar and Remm, 2007; Untergasser et al., 2012). Secondary structures were further assessed using OligoAnalyzer 3.1 (IDT, Integrated DNA Technologies, Coralville, IA, United States). Final oligo sequences were analyzed for strain-specificity using the BLASTn algorithm (National Center for Biotechnology Information, Bethesda, MD, United States). Primers and HPLC-purified, double-quenched probes (**Table 1**) obtained from IDT were reconstituted with TE to make a stock solution of 100 μM then stored at -20°C until use.

**Table 1:** Primers and probes for Bl-04 and NCFM.

Strain	Primers and probes^∗^	Amplicon Length (bp)	Concentration mM/ml
Bl-04	F: 5′-CTTCCCAGAAGGCCGGGT-3′	98	0.6
	P: 5′-6-FAM/CGAAGATGA/ZEN/TGTCGGAACACAAACACC CGG/3IABkFQ-3′		0.3
	R: 5′-CGAGGCCACGGTGCTCATATAGA-3′		0.9
NCFM	F:5′-CCACGACCAGATGTAACCAA-3′	209	0.6
	P: 5′-/6-HEX/TAAGCCGAA/ZEN/CAATGCTGAAACGAT/3IABkFQ-3′		0.3
	R: 5′-TTAGAAGATGCCAACGTCGAG-3′		0.9


**^∗^F, forward primer; R, reverse primer; P, probe.**

Primer annealing temperatures were optimized by testing a ±6°C temperature gradient from the labeled melting temperatures with 5Prime MasterMix Polymerase (5Prime, Gaithersburg, MD, United States) and a Mastercycler Gradient (Eppendorf, Hauppauge, NY, United States). Primer concentrations were optimized as outlined previously (Bustin, 2004) with a LightCycler^®^ 480 (Roche) and using SYBR Advantage^®^ qPCR Premix (Clontech, Mountain View, CA, United States). A probe concentration gradient was measured with Ex Taq^®^ DNA Polymerase (Clontech) on the LightCycler. All reactions used PCR-grade water (Teknova, Hollister, CA, United States).

### Quantitative PCR (qPCR)

**FIGURE 2:**
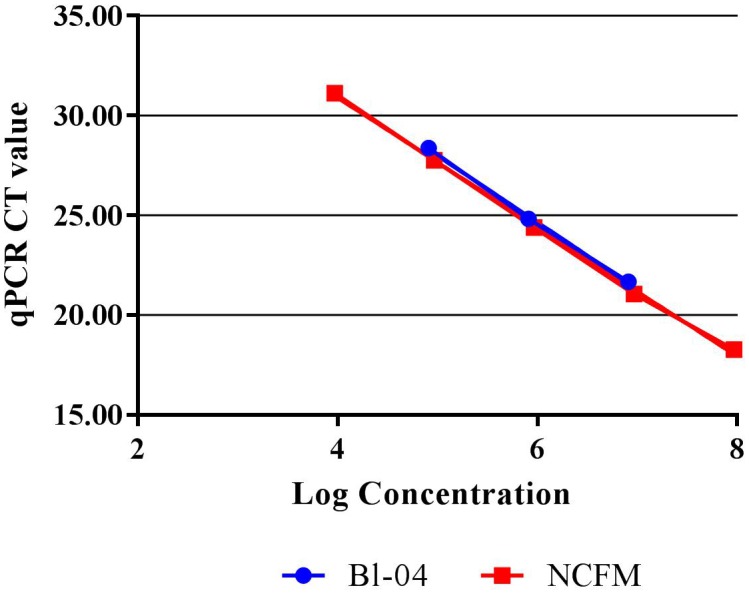
Standard curves of Bl-04 and NCFM gDNA from overnight culture through several dilutions using qPCR to measure threshold cycle (*C*_T_).

Quantitative PCR with the use of a standard curve was used to set the correct annealing temperature and primer concentration for each primer set, by determining the amplification efficiency and correlation coefficient. DNA was purified from approximately 1E+08 CFU per strain, using a Roche High Pure Template kit (Roche), and serially diluted to approximately 1E+04 CFU. Each 20 μl reaction mix included 10 μl SYBR^®^ Advantage^®^ qPCR Premix (Clontech, 639676), an optimized concentration of forward and reverse primers (**Table 1**) and either 2 μl of template DNA or 2 μl of sterile water for a no-template control. Samples were run in triplicate on the Roche Lightcycler 480 (Roche). Amplification consisted of a 5 min initial denaturing step of 96°C, followed by 40 cycles containing two steps, 10 s at 95°C and 30 s at 60°C. A standard curve of 90–105% efficiency and *r*^2^ value of >0.98 efficiency was created from these dilutions (**Figure 2**).

### Pretreatments

Propidium Monoazide (PMA) (Biotium Inc., Hayward, CA, United States) was used at 20 mM as an extracellular DNA intercalater, inhibiting the amplification of DNA associated with dead probiotic cells. PMA was used by adding 1.25 μl/ml to the cell suspension, in triplicate (Nocker et al., 2007b). PMA treatment was done on 1.2 ml of an 18 h culture, which equated to approximately 2E+09 cells. Samples were mixed and incubated in the dark at room temperature for 5 min on a shaking platform set at 200 rpm to improve the PMA binding. The PMA was cross-linked by photo-induction by exposure to a UV light source (PMA-Lite, Biotium Inc.) for 15 min. PMA treated samples were further used in the mechanical cell lysis treatments to create DNA for digital PCR.

Several DNA liberation methods were evaluated for used to provide DNA for cdPCR from 18-h overnight cultures of Bl-04 and NCFM.

1. A commercially available column based purification kit from Qiagen, Qiagen DNeasy Blood and Tissue Kit run on the QiaCube machine (Qiagen) was evaluated for its repeatability, using 6 × 1 ml NCFM culture only, not pretreated with PMA. This provided purified DNA eluted into 100 μl elution buffer to be used as the template material in subsequent PCR reactions.
2. A chemical lysis consisted of adding 10 μl of 1 μM chicken egg white lysozyme (Sigma-Aldrich) solution to 1 ml of culture, pretreated with PMA, and incubating for 15 min at 37°C before adding cell lysate as template material to the subsequent PCR reactions.
3. The bead beating mechanical disruption method included 1 ml 18-h overnight culture, pretreated with PMA, transferred to a 2 ml screw cap tube containing 250 μl of 0.1 mm zirconia/silica beads (BioSpec Products, Bartlesville, OK, United States). The tubes were agitated for 3 min at minimum speed in the 8 well bead beater (BioSpec Products). After 3 min, tubes were then incubated on ice for 1 min to cool the culture before a second 3 min agitation. This cell lysate was then added as the template material to the subsequent PCR reactions.
4. The second mechanical disruption method tested was sonication. One ml of an 18-h overnight culture, pretreated with PMA, was added to 9 ml diluent in a 15 ml screw top Falcon tube was placed on ice. The sonication wand (Thermo Fisher) was place 1 cm below the liquid surface and was allowed to sonicate for 5 min at 120 watts and 20 kHz. This cell lysate was then added as the template material to the subsequent PCR reactions.

### cdPCR

**FIGURE 3:**
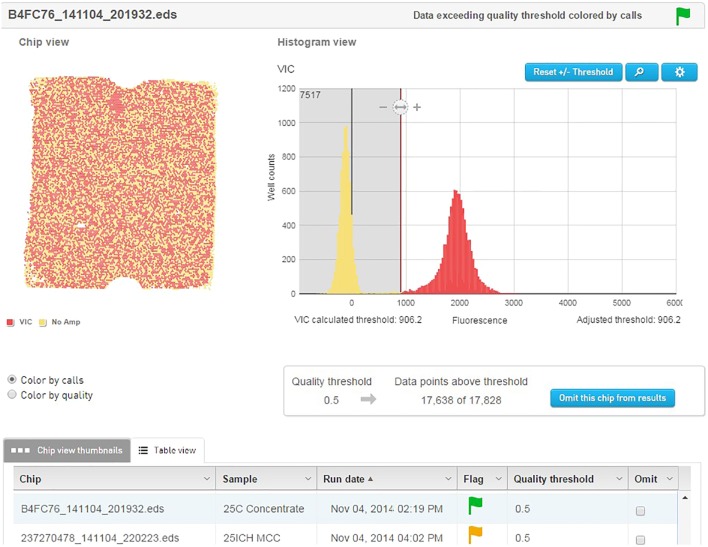
QuantStudio^®^ 3D Analysis Suite software example of a NCFM sample with good separation between the negative and positive results.

The template material was quantified and detected using an Applied Biosystems QuantStudio^®^ 3D digital PCR instrument (Life Technologies). Dilutions of template were prepared to approximately 2.0E+07 genetic copies per ml. This accounts for 75% coverage of the 20000 well chips after sample is further diluted by Mastermix. The PCR reactions were prepared to a final volume of 20 μl and contained 10 μl QuantStudio 3D digital PCR Master Mix, 2.4 μl of the forward primer (0.6 μM), 3.6 μl of the reverse primer (0.9 μM), 1.3 μl of probe (0.2 μM), and 1 μl of the cell dilution with the remainder being sterile ddH_2_O. The no template control (NTC) reactions replaced the 1 μl of cell dilution with additional sterile ddH_2_O. Of the 20 μl of the PCR reaction mix, 14.5 μl was applied to the QuantStudio^®^ 3D dPCR V2 20K chip using the QuantStudio^®^ 3D digital PCR chip loader, according to manufacturer’s instructions. The amplification was performed on the ProFlex 2x Flat PCR system, using an adjusted preset template modified to correct for underlying ramp settings. After amplification, the chips were inserted into the QuantStudio^®^ 3D chip reader for analysis and the data were transferred to the absolute quantification module contained on the cloud-based QuantStudio^®^ 3D Analysis Suite software. The threshold for the quality was set at 90% with a 95% confidence level. Results of each chip were visually analyzed on the software to ensure the default threshold was sufficient in separating the positive from the negative wells (**Figure 3**).

For dynamic range and limit of quantification (LOQ) evaluation, the number of genetic targets were calculated based on a survey of initial readings for the highest concentrated sample and theoretical dilutions were calculated. There is currently no reference standard for this type of testing. An initial DNA sample was diluted two-fold 8–9 times and seven dilutions were strategically selected for enumeration by cdPCR. DNA concentrations were then compared to the theoretical calculated concentrations. A one-way ANOVA was employed to investigate statistical differences (Minitab, Minitab Inc., State College, PA, United States). Samples with *P*-values of 0.05 were considered statistically different. Data are expressed as mean and standard deviation (SD) of triplicate measures determined in several independent experiments.

### Plate Count Enumeration

Lyophilized culture concentrate was enumerated on MRS agar (BD Difco, Sparks, MD, United States) with 0.05% cysteine (Sigma-Aldrich, St. Louis, MO, United States) at 37°C for 72 h under anaerobic conditions. Briefly, 11 g of concentrate were diluted in MRS broth (BD Difco, Sparks, MD, United States) at room temperature for 30 min. Samples were masticated for 1 min at 240 RPM before and after 30 min rehydration time before being serially in premade peptone dilution blanks (3M Microbiology Products, St. Paul, MN, United States) diluted to reach an individual plate count of 25 to 250 colonies.

## Results

### Specificity and Efficiency

**FIGURE 4:**
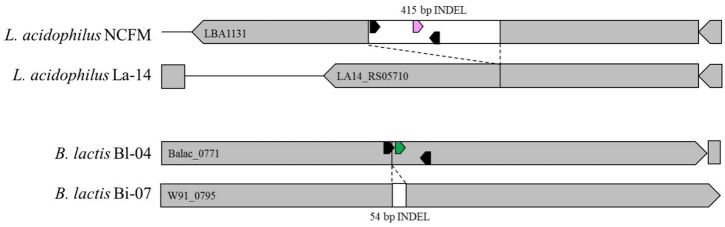
*In silico* representation of primer locations within INDEL regions of Bl-04 and NCFM relative to similar strains.

**FIGURE 5:**
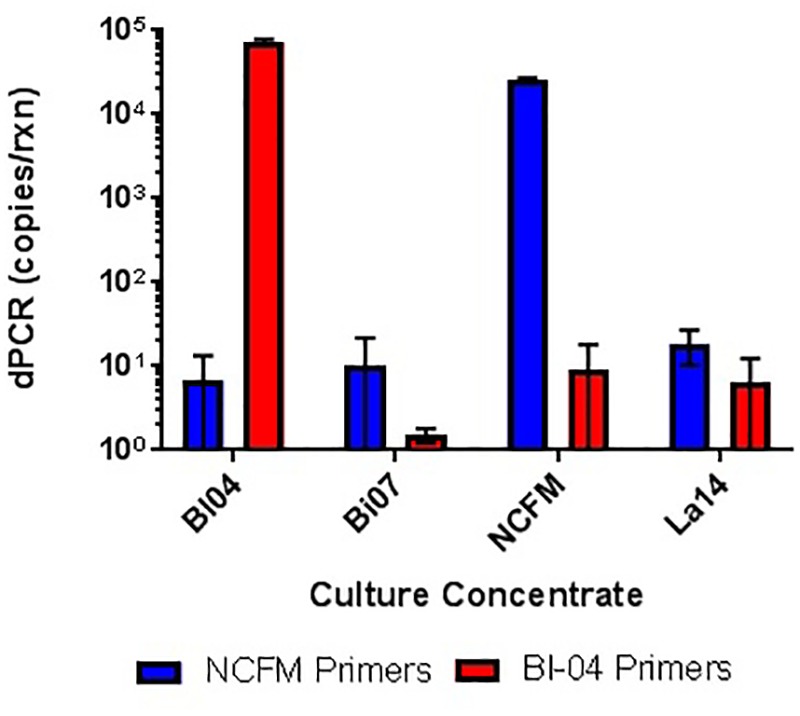
*In vitro* testing of lyophilized culture, pretreated with PMA, to show primer specificity of strain-specific NCFM and Bl-04 primers when tested against distantly and closely related strains.

The specificity (presence and amplification of the specific sequence of the target strain) of the primer sets was evaluated by the analysis of all fully sequenced similar species and strains of other taxa that were available within the private collection. The evaluation consisted of *in silico* (**Figure 4**) and *in vitro* testing (**Figure 5**), to rule out cross reaction with the primer sets to any similar strains.

Refinement of annealing temperatures and primer concentrations improved the detection efficiency of the PCR reactions. For initial experiments, an annealing temperature gradient (52–62°C) was used to cover a wide temperature range. Optimal primer set annealing temperature for NCFM was 54°C and Bl-04 was 60°C (data not shown). Various primer concentrations were tested for optimal amplification, with 0.6 mM forward and 0.9 nmM reverse primers preforming the best (data not shown). qPCR was used to assess the efficiency of the primer set on its ability to detect DNA extracted from pure culture. A dilution series was used to set up a standard curve and a linear regression was performed. NCFM had an *R*^2^ value of 0.9987 and an efficiency of 103% and Bl-04 had an *R*^2^ value of 0.9987 and an efficiency of 99% (**Figure 2**).

### Dynamic Range, LoQ and LoD

The theoretical range of digital chip PCR is based on the number of wells filled and analyzed. A chip has 20,000 wells and a range of 5–90% saturation is recommended to have a confidence interval of 95%. Therefore, the theoretical range is 1,000 to 18,000 loaded wells is translated into 1.4E+03 to 2.5E+04 genetic copies per μl because of the dilutions of the PCR reaction volume (20 μl) and the amount added to the chips (14.5 μl). The actual measured dynamic range for NCFM was 288–30,136 copies/μl and Bl-04 was 391–42,512 copies/μl using Poisson modeling (**Tables 2**, **3**).

**Table 2:** Bl-04 chip results, evaluating a dilution series of theoretical DNA target counts with measured counts from cdPCR.

Target copies/μl	# of Chips	cdPCR copies/μl	Average	*SD*	%RSD	Chip Precision	Number of wells qualified	Number positive wells
42,512	3	41,546	42512	2458	6%	1.97%	16,079	12,655
		40,684				1.84%	18,091	14,160
		45,307				1.93%	16,760	13,666
21,256	2^a^	20,558	21098	763	4%	2.08%	17,967	9,660
		21,637				2.15%	16,361	9,094
16,028	3	9,747	9870	188	2%	2.72%	17,694	5,430
		10,086				2.65%	17,999	5,690
		9,776				2.75%	17,250	5,309
2,657	3	2,024	1974	107	5%	5.82%	16,337	1,201
		2,047				5.95%	15,531	1,154
		1,852				6.00%	16,843	1,135
664	3	594	601	19	3%	10.62%	16,228	377
		623				10.63%	17,016	377
		587				11.40%	15,074	330
332	3	425	446	38	8%	12.45%	16,898	309
		423				12.27%	17,551	279
		489				11.80%	18,133	287
166	3	388	391	2	1%	12.82%	16,489	242
		393				13.49%	18,138	264
		392				13.43%	16,308	240
NTC	3	312	303	24	8%	14.83%	17,167	201
		276				16.07%	16,677	173
		322				14.99%	16,288	197


*^*a*^One of the three replicate chips did not load correctly, resulting in no read.*

**Table 3:** NCFM chip results, evaluating a dilution series of theoretical DNA target counts with measured counts from cdPCR.

Target copies/μl	# of Chips	cdPCR copies/μl	Average	*SD*	%RSD	Chip Precision	Number of wells qualified	Number positive wells
30,136	1^a^	30,201	N/A	N/A	N/A	1.98%	16,653	11,285
15,068	3	16,083	16487	783	5%	2.30%	17,128	7,763
		17,390				2.34%	16,738	7,891
		15,989				2.27%	17,524	7,914
7,534	3	7,704	7380	457	6%	3.09%	16,723	4,207
		7,057				3.21%	16,654	3,889
		7,268				3.13%	17,102	4,090
1,883	3	1,620	1722	144	8%	6.32%	17,265	1,024
		1,823				6.08%	16,606	1,104
		1,634				6.22%	17,661	1,056
942	3	909	900	13	1%	8.58%	16,814	567
		891				8.66%	16,851	557
		794				8.97%	17,647	521
471	3	503	472	44	9%	11.64%	16,852	317
		441				12.07%	17,946	296
		610				10.21%	17,884	407
235	3	287	288	1	0%	16.17%	15,851	171
		289				15.32%	17,448	189
		306				15.32%	16,457	189
NTC	3	182	141	122	87%	19.59%	17,520	120
		237				18.09%	15,634	139
		3				299.85%	17,515	2


*^*a*^Two of the three replicate chips did not load correctly, resulting in no read.*

The most critical measurement determination is for the lower limit of quantification (LoQ), or the lowest, most accurate count obtainable. The parameters were set to a maximum 10% precision program readout, 15% or lower %RSD and total of more than 10,000 qualified wells (both positive and negative) per chip, with an average of 900 copies/μl for NCFM and 1,974 copies/μl for Bl-04 (**Tables 2**, **3**).

**FIGURE 6:**
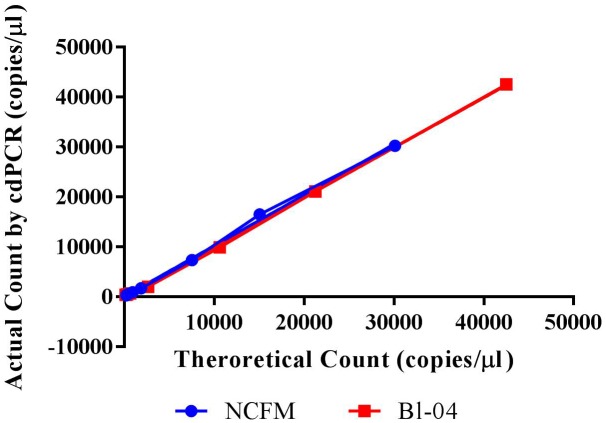
Chip-based digital PCR quantification of Bl-04 and NCFM gDNA as compared to the theoretical count.

The limit of detection (LoD) was defined as values above the no template control, which is also used to determine the false positive reactions. There are very low false positives associated with each assay, 0.52% for NCFM and 1.14% for Bl-04 (**Tables 2**, **3**), allowing for the LoD for NCFM to be 141 copies/μl and 303 copies/μl for Bl-04. This dynamic range was then used to evaluate the efficiency of the primer sets under cdPCR conditions for both strains, NCFM was 101% and Bl-04 was 100% (**Figure 6**) efficient, with *r*^2^ values over 0.99.

### Pretreatments

#### DNA Purification via Commercial Kits

**FIGURE 7:**
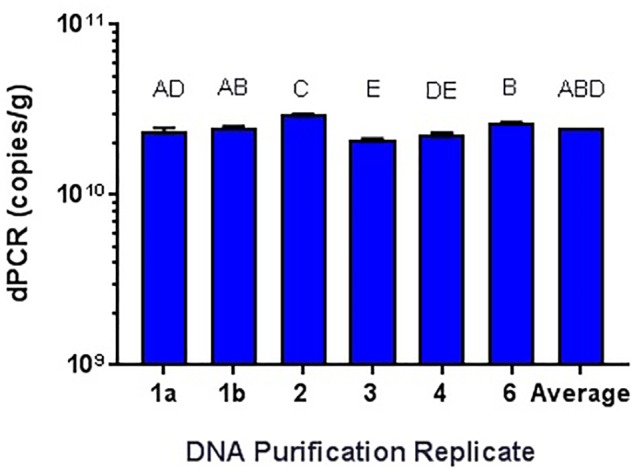
Qiagen DNeasy replicate isolations of DNA from NCFM overnight cells not treated with PMA. Columns with differing letter are significantly different from each other.

While it has been shown that kits developed for the isolation and purification of DNA are not 100% efficient (Mumy and Findlay, 2004; Wang et al., 2016), the variability needed to be tested. Using the Qiagen DNeasy Blood and Tissue kit on the Qiagen Qiacube system, six purifications of the same *L. acidophilus* NCFM overnight culture was performed, with significantly differing results (**Figure 7**). All samples were enumerated using cdPCR and were compared to the average value. Sample 5 did not amplify at all and was not included in the comparison. There was one sample (Sample 3) that was significantly higher than the average and there was an average 12% RSD between all samples. There was no difference shown when a single sample (Sample 1) was enumerated with two separate replicates (a and b).

##### PMA treatment

**FIGURE 8:**
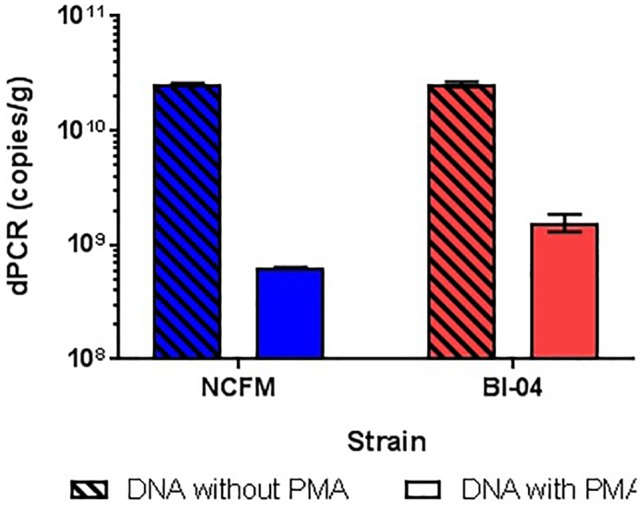
Inhibition of cdPCR by treatment with PMA on Bl-04 and NCFM gDNA.

The amplification of DNA associated with dead and highly damaged cells can cause artificially high cell counts, so experiments were conducted to determine the best way of inhibiting this amplification. gDNA from overnight broth cultures of NCFM and Bl-04 not treated with PMA was isolated and purified with Qiagen DNeasy Blood and Tissue kit was used as a representative for the dead cells, as the simplest possible model. PMA was found to hinder DNA amplification over one log when compared to DNA not treated to PMA (**Figure 8**). This has been previously reported in literature (Desfossés-Foucault et al., 2012).

##### DNA liberation

**FIGURE 9:**
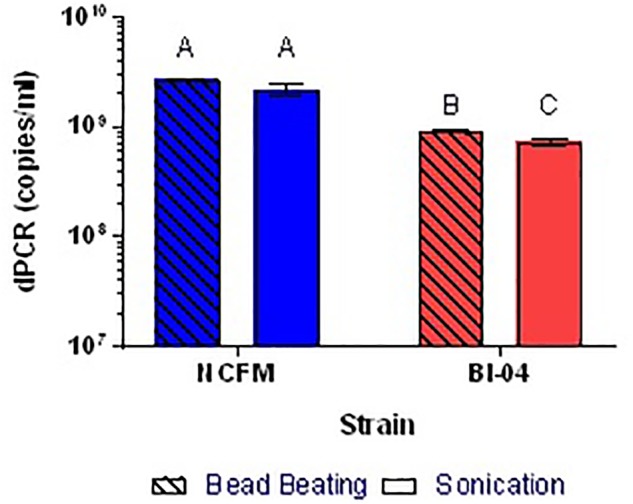
Comparison of bead beating and sonication mechanical DNA liberation methods on Bl-04 and NCFM from overnight cells pretreated with PMA. Differing letters represent significant differences between methods.

Because the SEM (**Figure 1**) allowed visualization of the size of the cell compared to the well, cell membrane material will likely not block DNA from entering the chip wells. The wells within the chip are 60 μm in diameter whereas *Lactobacillus* and *Bifidobacterium* less than 10 μm, thus purification of the template DNA is not required. Liberation of DNA from cells with an intact membrane was optimized by comparing two mechanical methods. Lysis by chemical means was attempted but inhibited the end-point PCR (data not shown). Sonication and bead beating were both applied to PMA pretreated cells from broth culture, with varying success but it was clear that bead beating increased the available DNA for Bl-04 (**Figure 9**) and was less variable than sonication (3% vs. 9%) for both cultures.

#### Applicability

A two sample *T*-test was used to compare two technicians’ reproducibility within an assay and a single technician’s reproducibility between assays. ANOVA analysis demonstrated no differences detected either between technicians or between runs for either strain for gDNA (**Tables 4A,B**). However, there were differences detected between assays, but because that difference is so small as compared to the plate count enumeration variability, it was concluded that the reproducibility of the cdPCR method was satisfactory for the remainder of the study.

**Table 4:** Repeatability and reproducibility assays for Bl-04 **(A)** and NCFM **(B)** as measured in repeated Master Mix (MM) set up.

	MM1	MM2	MM3	MM4	MM5	Repeatability series
**(A)**

Average copies per Master Mix	16,204	17,149	16,542	17,931	17,166	16,998
Stdev copies per Master Mix	695	556	1041	605	482	676
%RSD	4%	3%	6%	3%	3%	4%
Highest Chip	17,033	17,896	17,350	18,429	17,786	18,429
Lowest Chip	15,406	16,339	14,950	16,787	16,541	14,950

**(B)**

Average copies per Master Mix	52,481	48,235	54,202	51,364	43,455	49947
Stdev copies per Master Mix	4061	3923	2304	2949	853	2818
%RSD	8%	8%	4%	6%	2%	6%
Highest Chip	58,459	55,135	56,372	56,098	44,773	58,459
Lowest Chip	47,715	44,303	50,232	48,043	42,800	42,800

When assessing the specificity of cdPCR, DNA from NCFM and Bl-04 were mixed at a 50/50 ratio (v/v). Individual strain DNA (diluted by half, v/v) and mixed DNA samples were run in multiplex and in single reactions to determine the accuracy of the assay. When NCFM DNA was run at 54°C with NCFM primers, the count was 1.29E+09 copies/ml vs. 1.18E+09 copies/ml when NCFM DNA was run at 54°C with multiplexed primers. Results were similar when the mixed DNA sample was run with multiplexed primers at 54°C, 1.10E+09 copies/ml. However, when the mixed DNA sample was run with multiplexed primers at 60°C the results were significantly lower than the previously reported results, 1.03E+09 copies/ml.

**FIGURE 10:**
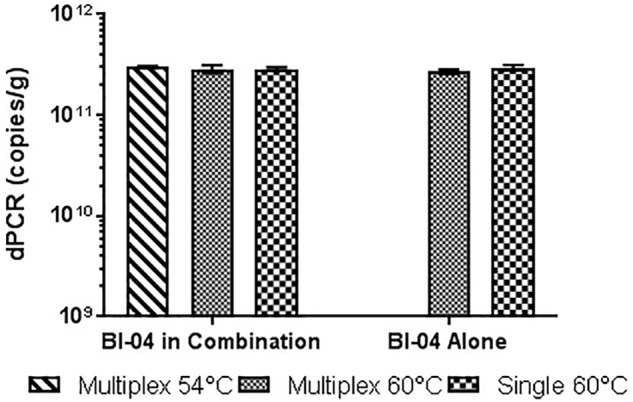
Lyophilized Bl-04 culture, pretreated with PMA and bead beat, amplified singly and in combination (containing Bl-04 and NCFM) at two difference temperatures with single target primer master mix or multiplexed primer master mix. No significant differences seen between samples.

**FIGURE 11:**
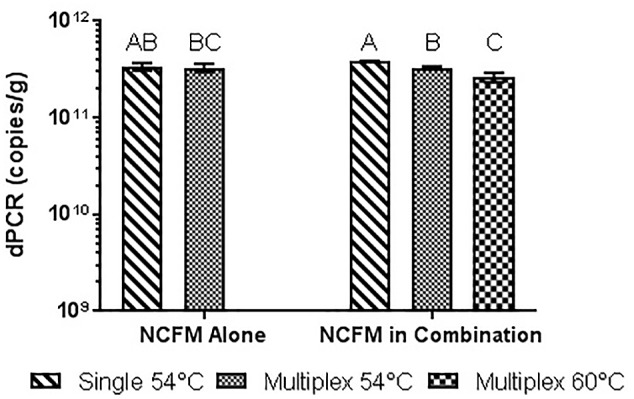
Lyophilized NCFM culture, pretreated with PMA and bead beat, amplified singly and in combination (containing Bl-04 and NCFM) at two difference temperatures with single target primer master mix or multiplexed primer master mix. Columns with differing letter are significantly different.

Similar tests were run detecting the Bl-04, with no significant differences detected when run at 54°C vs. 60°C or when run with Bl-04 specific primers or multiplexed. This comparison was tested against lyophilized culture, again with no significant differences seen in the Bl-04 reactions, but the NCFM sample run at 60°C was significantly lower than its counterparts (**Figures 10**, **11**).

Power curve calculations were done (Mini-tab, Powered for Pair t) comparing the %RSD of the various measurement systems available for bacterial enumeration. With a variation of 20–30%, CFU was calculated using a 15% RSD, and FCM a 10% RSD determined from Bl-04 and NCFM runs (Data not shown). In order for both CFU and FCM to achieve the same detectable difference as cdPCR (at 5%RSD) it would require 17 and 9 replicates, respectively (**Table 5**).

**Table 5:** Power Curve analysis showing the number of replicates needed by various measurement systems to be comparable to the % detectable difference that cdPCR can achieve.

Method	Iterations	%RSD	% Detectable difference
Plate Count	3	15.0%	46.0%
	17	15.0%	15.0%
	25	15.0%	12.2%
cdPCR Bl-04	3	4.0%	12.2%
cdPCR NCFM	3	5.0%	15.0%
Flow Cytometry	3	10.0%	30.8%
	9	10.0%	15.0%
	11	10.0%	12.2%


**FIGURE 12:**
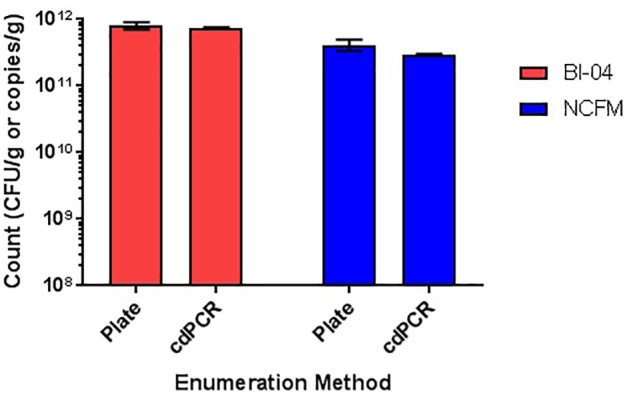
Enumeration of commercially manufactured lyophilized culture concentrate via plate count enumeration compared to strain-specific cdPCR.

Comparison of plate count enumeration to cdPCR on lyophilized commercially available culture concentrate was completed. The final cdPCR method used for this comparison included PMA treatment and bead beating for DNA liberation. While cdPCR results (7.55E+11 copies/g for Bl-04 and 2.94E+11 copies/g for NCFM) were slightly lower than plate count (7.7E+11 CFU/g for Bl-04 and 4.14E+11 CFU/g for NCFM), they were not significantly lower for either strain (**Figure 12**). The actual %RSD for the plate count of BL-04 was 9% and 19% for NCFM while the %RSD for cdPCR of Bl-04 was 4% and 3% for NCFM.

## Discussion

Increased research and consumer awareness of the various benefits of probiotics will contribute to the expected $50 billion global industry by 2020 (Probiotic Ingredients Market worth 46.55 Billion Usd by 2020, 2017). Current regulatory requirements are claim dependent (U.S. Food & Drug Administration, 2008; Matulka, 2016; BeBest Practices Guidelines for Probiotics | Council for Responsible Nutrition, 2017) but in order to reference studies for which claims are made, strain identification and quantification of individual strains within multi-strain products (Kolaček et al., 2017) will be imperative to protect consumers from product adulteration.

Establishment of new enumeration methods for lyophilized cultures can help overcome some of the current limitations with microbial based enumeration in the probiotic industry. Although no known method can completely satisfy all the needs of this industry, the described methods of cdPCR contribute significant improvements upon current technology. Digital PCR, in general, has short time to results and less variability than current methods. In commercial laboratories, CFU method includes duplicate or triplicate sampling and assumes a %RSD of 15 to 30% (or more). Testing based on microbial plate counts should utilize statistical power analysis to determine the number of replicates but is sometimes forgone in lieu of time and cost effectiveness in routine testing. cdPCR is considerably less time consuming and has lower method variability, allowing for sampling to be done properly for the required statistical power.

Another key advantage of cdPCR is the detection of a strain-specific DNA target, which can be easily developed for any strain that has a sequenced genome. Single-copy genetic targets, each representing a single cell, are sufficiently separated in chip wells to provide absolute enumeration without the use of a standard curve. Amplification of genetic targets associated with damaged cells or present extracellularly can be inhibited by DNA- intercalating dyes, such as PMA. It is unknown how damaged a cell needs to be in order for PMA to intercalate, so this method may not reliably enumerate samples containing sub-lethally injured cells (Nocker et al., 2007a). Conversely, PMA may allow amplification of DNA associated with dead cells that have an intact membrane, which could be an issue in temperature abused stored samples (Kramer et al., 2009; Davis, 2014). While this subset of cells could influence the application of this method on stored samples, it is difficult to determine if heat or chemical treatments are actually creating this population or allowing the cells to go into an inactive, dormant state. Further work needs to be done to produce and evaluate these cells.

Current CFU methods rely on growth using general and selective media which only detect bacteria that are able to divide and create a colony. This does not technically satisfy the WHO definition of a probiotic, which is “live micro-organisms when administered in adequate amounts, confer a health benefit on the host”. The term “live” needs to be further defined beyond simply replicating to include specific metabolic functions not associated with cell division and it has been suggested that the definition of a probiotic include populations that are metabolically active and/or have intact membranes (Ouwehand and Salminen, 1998; Davis, 2014; Hill et al., 2014) because it has been discovered that even dead cells may have some beneficial effects (Lahtinen, 2012). Specific probiotic strains are selected for their ability produce health benefits in a human environment, which many have been isolated from and may never fully adapt to grow in a synthetic environment (Mills et al., 2011) and it is unclear how the synthetic growth medium affects the cell population that is being enumerated. Plate count measurements are not able to count the cells that are viable but not dividing or are not cultureable (VBNC), which may underreport the number of cells (Lahtinen et al., 2008; Volkert et al., 2008). Conversely, this also does not account for cells that have sub-lethal damage to their cell membranes and may be able to recover and replicate on specialized media, but when introduced to harsh or stressed environments like the human gut, may not be able to survive to confer those same health benefits.

One other important issue with CFU is the inability to identify specific strains from one another, which is a major short coming with products that contain genetically similar bacteria. The ability to administer a specific strain at an accurate cell count is the basis on which clinical trials base their recommendations. Trials have been conducted where outcomes and or benefits can then be associated with structure/function health claims. While expert panels agree that probiotic effects are strain-specific (Hill et al., 2014), recommended method for strain taxonomy is not readily defined in the literature (Foerst and Santivarangkna, 2015). The historic definition in *Bergey’s Manual of Systematic Bacteriology* was “A strain is made up of the descendants of a single isolation in pure culture and usually is made up of a succession of cultures ultimately derived from an initial single colony” (Staley, 2006), which focuses on phenotypic relatedness. There are now many general and high resolution methods for analyzing the genotypes of different strains (MacCannell, 2013) that, when combined with phenotypic analysis, show that a single SNP can have a measurable effect on the function and efficacy of cells (Briczinski et al., 2009). Furthermore, many bacterial taxa are so similar that they can only be distinguished at the SNP level (Achtman, 2008; Lomonaco et al., 2015). Digital PCR can be used to distinguish and quantify very similar strains of probiotics using deletions in genes, but this technology can theoretically be used to selectively detect organisms differing by a single SNP.

The results show how applicable cdPCR could be to absolutely enumerate probiotic cells at the strain level for the dietary supplement industry, when the strain’s genome is sequenced. This technology is more sensitive for strain specific detection with less variability than the current standard of plate count enumeration, without the results being significantly different between the two methods. This is the first cdPCR method developed for the absolute enumeration of probiotics and is a good starting point for the development of additional methods for stored and stressed dietary supplements, which have complications beyond what has been addressed here. Beyond dietary supplements, additional applications could include probiotic enumeration in food, but those matrixes may also provide complications and new pretreatments may need to be implemented and are not addressed in this initial proof of concept study. Future work could also include evaluation of additional DNA-intercalating dyes to determine cell viability. For this method to be employed in routine testing, it would have to be properly validated via inter and intra-laboratory trials to confirm its efficiency at the strain level.

## Author Contributions

WM and BS developed the quantitative assays and SH implemented cdPCR. SH and MD performed the optimization experiments. CS and BS performed the project management. All authors contributed to the manuscript writing.

## Conflict of Interest Statement

All authors are employees of DuPont Nutrition & Health, which commercializes the probiotic strains used in the experiments.
